# Astellas article: UK multidisciplinary recommendations on use of combination first-line enfortumab vedotin▼ and pembrolizumab in advanced urothelial carcinoma

**DOI:** 10.1093/oncolo/oyaf220

**Published:** 2025-11-10

**Authors:** Syed Hussain, Aisling Carr, Simon John Crabb, Vikash Dodhia, Louise Fearfield, Jahangeer Mahmood Malik, Maria Lapuente, Davina Lau, Harry Petruskin, Naveed Sarwar, Nicola Jane Smith, Robert Stevenson, Robert Jones

**Affiliations:** School of Medicine and Population Health, Department of Oncology, University of Sheffield, Sheffield S10 2SJ, United Kingdom; School of Cancer Sciences, University Hospital Southampton NHS Foundation Trust, Southampton SO17 1BJ, United Kingdom; National Hospital for Neurology and Neurosurgery (NHNN), University College London Hospitals NHS Trust, London W1T 4JD, United Kingdom; Mount Vernon Cancer Centre , East and North Hertfordshire NHS Trust, Northwood WD3 1PZ, United Kingdom; Department of Dermatology, Chelsea and Westminster NHS Hospital Trust, London SW10 9NH, United Kingdom; Edinburgh Cancer Centre, NHS Lothian, Edinburgh EH4 2XU, United Kingdom; Barts Cancer Research Centre, St Bartholomew’s Hospital, London EC1A 7BS, United Kingdom; Astellas Pharma Ltd, Addlestone KT15 2NX, United Kingdom; Moorfields Eye Hospital, London EC1V 2PD, United Kingdom; Imperial College Healthcare NHS Trust, London SW7 2BX, United Kingdom; HCA Healthcare UK, London NW1 2BU, United Kingdom; University Hospitals Birmingham NHS Foundation Trust (UHB NHS FT), Birmingham B15 2GW, United Kingdom; School of Cancer Sciences, University of Glasgow, Glasgow G12 0YN, United Kingdom

**Keywords:** bladder cancer, enfortumab vedotin, pembrolizumab, adverse events, consensus, multidisciplinary team

## Abstract

**Prescribing information & ADR reporting:**

This is an Astellas article. Prescribing information can be accessed at https://dhgd52pup2eiv.cloudfront.net/23ffe42b-4e89-48e1-b8d8-08b726ed94c1/b5d6cdf5-d05a-4bb1-9965-de9a244d39eb/b5d6cdf5-d05a-4bb1-9965-de9a244d39eb_source__v.pdf and the adverse event reporting information can be found on the last page of that link.

**Background:**

Advanced urothelial carcinoma (UC) significantly impacts quality of life, is associated with poor prognosis, and carries a high economic burden. Recently, the combination of enfortumab vedotin and pembrolizumab (EV-P) has demonstrated improved progression-free survival vs platinum-based chemotherapy and overall survival, and is now recommended as a first-line therapy for patients with unresectable or metastatic disease who are platinum eligible.

**Methods:**

A multidisciplinary expert panel was convened to review the current UK patient pathway for advanced UC. The panel developed consensus recommendations for implementing EV-P in the United Kingdom and provided guidance on managing adverse events (AEs), taking into account the challenges in the current pathway.

**Results:**

The expert panel recommended leveraging lessons from the previous implementation of new immunotherapies and antibody-drug conjugates as EV-P is implemented across the United Kingdom. They emphasized the importance of peer support from clinical centers involved in the EV-302 phase 3 clinical trial, advocating for the sharing of protocols, advice, and support for toxicity management. Recommendations included establishing robust referral pathways and multidisciplinary care models tailored to the resources and structures of different hospital settings. Education in proactive side effect identification and management was recommended for bladder cancer clinical nurse specialists, acute oncology nurses, pharmacists, and clinicians. The panel developed patient checklists to support clinicians in assessing treatment suitability, monitoring AEs during therapy, and ensuring continued monitoring after treatment ends. Detailed recommendations were provided for managing AEs, with a focus on skin reactions, peripheral neuropathy, hyperglycemia, pneumonitis/interstitial lung disease, and ocular disorders, along with guidance on when to involve specialist services.

**Conclusion:**

These consensus recommendations provide practical, multidisciplinary guidance to support the effective implementation of EV-P for advanced UC in UK healthcare settings.

## Astellas sponsored article

## Prescribing information & ADR reporting

This article was sponsored by Astellas Prescribing Information can be accessed https://dhgd52pup2eiv.cloudfront.net/23ffe42b-4e89-48e1-b8d8-08b726ed94c1/b5d6cdf5-d05a-4bb1-9965-de9a244d39eb/b5d6cdf5-d05a-4bb1-9965-de9a244d39eb_source__v.pdf. and the adverse event reporting information can be found on the last page of that link.

Implications for Practice To support the implementation of the enfortumab vedotin and pembrolizumab combination in the United Kingdom, an expert multidisciplinary panel developed consensus recommendations. Incorporating these recommendations into clinical practice will help ensure that patients who may benefit from this therapy can access it and that the multidisciplinary care team is adequately supported in managing adverse events (AEs). Recommendations for implementation include peer support between centers, multidisciplinary care models, establishing referral pathways, and education on side effect management. The expert panel also developed checklists for patient suitability and monitoring and provided guidance on managing AEs and determining when to refer to specialists.

## Introduction

Bladder cancer is the 11th most common cancer in the United Kingdom. Each year, approximately 10 500 new cases of bladder cancer are diagnosed, with around 5600 deaths.^1^ Bladder cancer is broadly categorized into non-muscle invasive bladder cancer (NMIBC), which is comprised of Ta, Tis, and T1 disease; muscle-invasive bladder cancer (MIBC), including T2, T3, and T4 with or without regional lymph node involvement; and metastatic disease (where there is distant spread). Locally advanced bladder cancer is a term generally used to include T3b, T4, and/or N1−N3 disease. NMIBC has a lower risk of metastasis than MIBC, but it can recur and/or progress to muscle-invasive disease.^2^

Although most patients have organ-confined disease at diagnosis, around 10% have unresectable metastatic disease.^3^ Approximately 50% of patients who undergo radical treatment for muscle-invasive disease relapse, in most cases with distant metastases.^4^ The 5-year overall survival (OS) rate in patients across all disease stages is 52.2%, with survival rates inversely correlated with disease stage.^4^^,^^5^ Urothelial carcinoma (UC) accounts for 90% of bladder cancers, with the remaining 10% having non-UC histology (ie, squamous, small cell, sarcomatoid, or adenocarcinoma).^6^ Metastatic UC is associated with a high economic burden driven by hospitalizations, emergency department (ED) visits, and end-of-life care. Pain associated with locally advanced/metastatic UC impacts physical and daily activities, and patients are further impacted by worsening physical and role functioning, pain, and overall quality of life as metastatic UC progresses.^6–12^ The prognosis of metastatic UC is poor, with a 5-year survival rate of just 12%.^13^

Systemic anticancer treatment is recommended for patients diagnosed with de novo or relapsed unresectable locally advanced or metastatic UC (termed advanced UC hereafter), with the aim of extending survival and improving symptom control.^6^^,^^14–16^ Until recently, cisplatin-based chemotherapy regimens were the standard of care as first-line therapy for patients fit enough to receive cisplatin. Carboplatin-based regimens were used for those unsuitable for cisplatin, with immune checkpoint inhibitors (ICIs) (atezolizumab or pembrolizumab in some jurisdictions) an option for patients with programmed death-ligand 1 (PD-L1)-positive tumors for whom chemotherapy is unsuitable. Platinum-based chemotherapy may be followed by avelumab maintenance therapy in those with ongoing clinical benefit at the end of chemotherapy, and pembrolizumab or atezolizumab is used as a second-line therapy in patients whose disease progresses during or after platinum-based chemotherapy.^4^^,^^6^^,^^14–16^

In the United Kingdom in 2021, 41.5% of patients diagnosed with stage 3 bladder cancer and 32.8% of those diagnosed with stage 4 disease received systemic anticancer therapy in the form of chemotherapy.^17^ Globally, real-world data show that around 40% of patients receive first-line systemic treatment, while only 15%-20% of advanced UC patients proceed to second-line or later treatments.^4^^,^^18–22^ Many patients are ineligible for currently available therapies, and there is a high attrition rate between the first- and second-line settings.

In 2024, 2 regimens were endorsed by the European Society of Medical Oncology (ESMO) and the European Association of Urology (EAU),^16^^,^^23^ which address the unmet need for more effective therapies. One is the antibody-drug conjugate (ADC) enfortumab vedotin in combination with ICI pembrolizumab, and the other is cisplatin and gemcitabine chemotherapy in combination with the ICI nivolumab.

**Figure 1.**
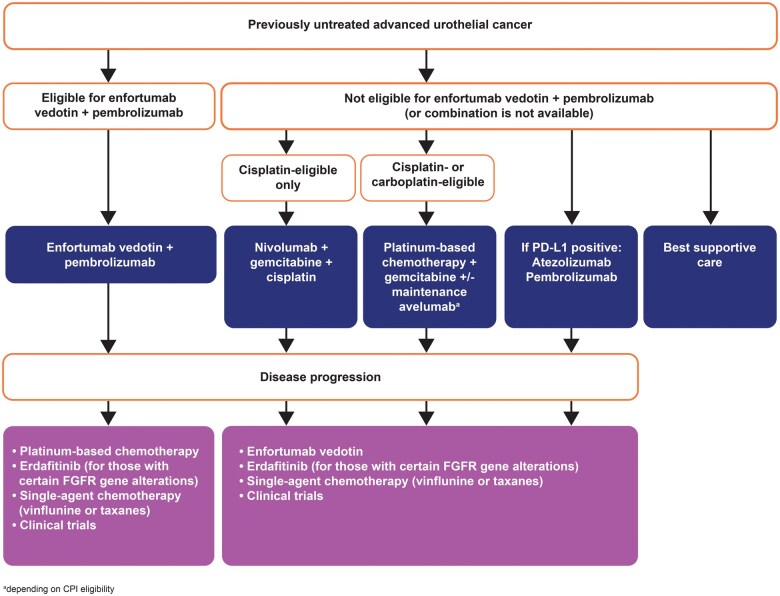
EAU and ESMO recommendations for the management of patients with advanced urothelial carcinoma.^15^^,^^23^ Abbreviations: CPI, checkpoint inhibitor; EAU, European Association of Urology; ESMO, European Society of Medical Oncology; FGFR, fibroblast growth factor; PD-L1, programmed death ligand-1.

Enfortumab vedotin and pembrolizumab (EV-P) was licensed for patients with untreated unresectable or metastatic UC based on the primary analysis of the EV-302 global, open-label, randomized phase 3 clinical trial, which demonstrated statistically significant improvements in progression-free survival (PFS) (median, 12.5 months vs 6.3 months; hazard ratio [HR] for disease progression or death, 0.45; 95% CI, 0.38-0.54; *P* < .001) and OS for EV-P (median, 31.5 months vs 16.1 months; HR for death, 0.47; 95% CI, 0.38-0.58; *P* < .001) compared to platinum-based chemotherapy.^24^ The EV-302 study also demonstrated a lower incidence of treatment-related adverse events (AEs) of grade 3 or higher in the EV-P arm than in the chemotherapy arm.^24^ The combination is now recommended as a first-line therapy for advanced UC in guidelines from ESMO, EAU, and the National Comprehensive Cancer Network (Figure 1).^6^^,^^16^^,^^23^ The combination is licensed in the European Union (September 2024) and in the United Kingdom (October 2024).

At the American Society of Clinical Oncology Genitourinary Cancers Symposium in February 2025, data from longer-term follow-up of a median 29.1 months were presented with a median PFS of 12.5 vs 6.3 months; HR, 0.48; 95% CI, 0.41-0.57; *P* < .00001.^25^ Additionally, the median OS from these data was 33.8 vs 15.9 months; HR, 0.51; 95% CI, 0.43-0.61; *P* < .00001, showing maintenance of benefit with enfortumab vedotin plus pembrolizumab vs chemotherapy. The benefit was seen across all prespecified subgroups, regardless of cisplatin eligibility.^25^

The confirmed objective response rate (ORR) benefit (67.5% vs 44.2%) with enfortumab vedotin plus pembrolizumab was maintained vs chemotherapy with longer follow-up, with a median duration of response of almost 2 years (23.3 months) in the enfortumab vedotin plus pembrolizumab group and 7 months in the chemotherapy group.^25^ At 24 months, the complete response rate was 74.3% for patients on enfortumab vedotin plus pembrolizumab and 43.2% for patients on chemotherapy.^25^

The combination of nivolumab plus gemcitabine-cisplatin chemotherapy received a European license in metastatic bladder cancer in May 2024 on the basis of the multinational, open-label phase 3 trial Checkmate-901, which demonstrated statistically and clinically significant improvement in OS (median, 21.7 months vs 18.9 months; HR for death, 0.78; 95% CI, 0.63-0.96; *P* = .02) and PFS (median, 7.9 months vs 7.6 months; HR for progression or death, 0.72; 95% CI, 0.59-0.88; *P* = .001) with a manageable safety profile.^26^ Based on these results, ESMO and EAU subsequently recommended nivolumab plus gemcitabine-cisplatin as the first-line standard of care treatment for patients with metastatic bladder cancer who are eligible for cisplatin but ineligible for the EV-P combination (Figure 1).^16^^,^^23^

As therapies are welcomed by the clinical community, we also face the challenge of translating these discoveries into practical frontline solutions in terms of implementation and side effect management, particularly as it has been observed at other tumor sites where ADCs are routinely used that the side effect profiles and dosing of each molecule in this class differ quite significantly from other treatments and from other ADCs.^27^ In addition, being able to reach all eligible patients in a timely manner while balancing the needs of the already overstretched workforce has been and remains an increasing challenge for oncology services. Therefore, a multidisciplinary expert panel was convened to gain insight into the current patient pathway in the United Kingdom for advanced UC and AE management and make recommendations to aid oncology services in implementing EV-P in the United Kingdom.

## Methods

**Figure 2.**
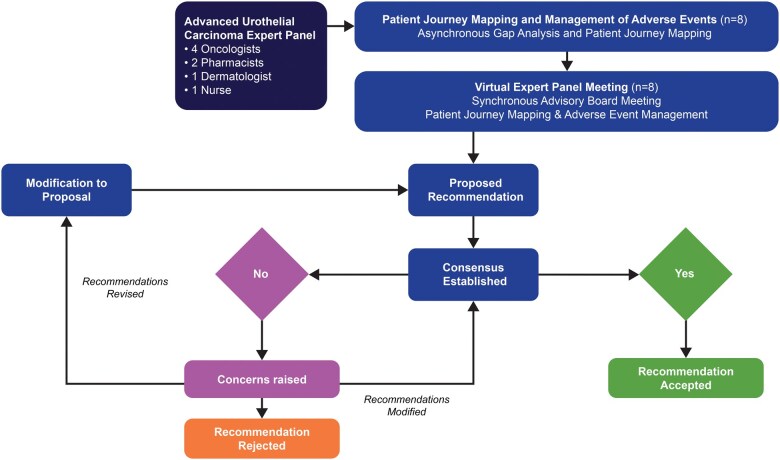
Consensus process.

A panel of medical experts engaged in a 2-part consensus activity. The experts were selected based on representation from both the public and private sectors, as well as representation of all the members of the multidisciplinary care team for advanced UC (Figure 2). The first part was an anonymized, asynchronous insight-gathering activity focused on understanding the patient journey through the lens of each care team member’s role, as well as a primer discussion on the approach to the management of AEs. The output of the activity was analyzed and summarized in an output report that was used as a pre-read for a full-day consensus meeting. The consensus meeting engaged the same expert panel and was facilitated by a medical oncologist panel member as chair. Consensus was achieved through a structured expert panel discussion, during which draft recommendations were reviewed, revised, and finalized through group deliberation and majority agreement. The analysis report from the consensus meeting and updated patient journey map were used to create a draft consensus paper that was reviewed individually by each panel member in a blinded review. Generative artificial intelligence technology was not used for any aspect of this work, including for the text, figures, tables, or any other content.

## Implementation of EV-P in the United Kingdom

### Current UK advanced UC pathway

**Figure 3.**
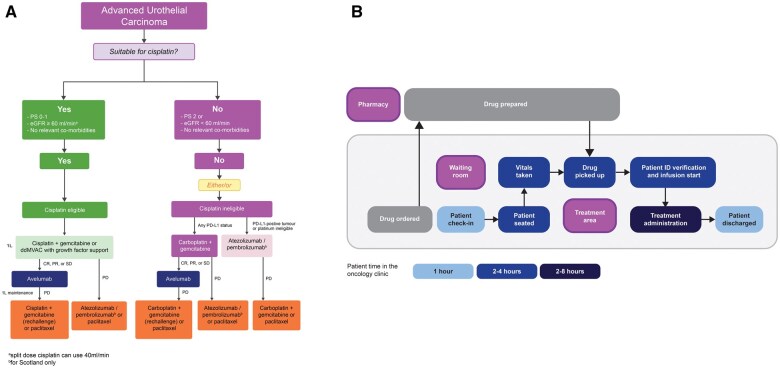
Current UK advanced urothelial carcinoma pathway. (A) Current management algorithm. (B) Flow diagram of treatment in the oncology clinic. Abbreviations: 1L, first-line; CNS, clinical nurse specialist; CR, complete response; dd, dose-dense; ED, emergency department; eGFR, estimated glomerular filtration rate; GP, general practitioner; MVAC, methotrexate, vinblastine sulfate, doxorubicin hydrochloride, and cisplatin; PD, progressive disease; PD-L1, programmed death ligand 1; PR, partial response; PS, performance status; SD, stable disease.

The current management algorithm for advanced UC is shown in Figure 3A.^4^ For patients with advanced disease, there is a need for a specialist multidisciplinary team assessment, with a discussion of appropriate treatment options and a discussion with the patient about their priorities.

Patient treatment in the oncology clinic typically follows a 2-step pathway where the drug is ordered, and drug preparation is planned in advance (Figure 3B). Immunotherapy drugs usually have long expiry dates and are flat-dosed, with an administration time of 1 to 2 hours, whereas cisplatin chemotherapy has a longer administration time of 4 to 8 hours. Other medications may have shorter expiry times compared to immunotherapy. There is variability across centers as to whether medications are prepared in aseptic units locally or in a designated treatment room area using a closed system device, or brought in pre-made. High-cost drugs are not always made in advance due to concerns about wasting medication if the pretreatment checks are not in place. Pretreatment toxicity assessment and blood/biochemistry tests are done either in the clinic or before the visit. Prescriptions must be clinically validated by a pharmacist. The frequency of follow-up appointments depends on the length of the cycle and the occurrence of side effects.

### Challenges with the current UK advanced UC pathway

There are multiple challenges and barriers to timely diagnosis and optimal treatment for patients with advanced UC in the United Kingdom, including delays to referral and diagnosis, patient suitability for treatment at the time of recurrence/progression or diagnosis of advanced disease, and limited access to second-line treatment options.^28^^,^^29^ Although this work focuses on the implementation of EV-P within the UK healthcare system, similar barriers may be present in other public healthcare systems.

Barriers to access to effective innovative therapies are diverse and include (1) patient factors (frailty, comorbidities); (2) delayed referral and protracted diagnostic pathways resulting in late review by oncology (eg, delays in trans urethral resection of bladder tumor); (3) patients who are not referred from a district general hospital to an academic or cancer center; (4) limited referral and/or access to specialist centers where innovative treatments are available; and (5) clinicians may underestimate the effectiveness of systemic anticancer therapy. Failures or delays in the referral pathway create barriers to timely care as well as to receiving the appropriate care for advanced disease. With the changing landscape of treatments now giving bladder cancer patients the opportunity to live longer, it is important that patients are given the opportunity to be seen by an oncologist and/or the multidisciplinary team to have their fitness assessed before a decision is made to refer them directly to palliative care. Assessment of patients’ suitability for systemic therapy by an oncologist is key before determining they are unfit for systemic treatment.

The expert panel noted that another barrier to receiving timely and appropriate treatment is that the entry point for care for advanced UC has shifted in the past few years, with more patients presenting to the ED. This may be because public education campaigns about hematuria are leading more patients to visit the ED. Patients may also present to the ED because of misdiagnosis by general practitioners, who may treat patients’ symptoms with multiple courses of antibiotics, thinking they have a urinary tract infection. Additionally, some patients may believe that they will receive quicker assessments and scans if they go to the ED instead of their general practitioner.^28^^,^^29^ Despite this, oncologists have seen more referrals for locally advanced disease than metastatic disease in the last 6 to 12 months.^30^

There are also challenges in providing optimal systemic anticancer treatment to patients. Poor awareness of the symptoms of bladder cancer in the general public may lead some patients to present late with advanced disease. In addition, more than half of cases of bladder cancer are diagnosed in patients over 75 years of age, who are more likely to have poor renal function, poor performance status, and multiple comorbidities, including significant cardiovascular risks.^1^ These factors render patients ineligible for systemic anticancer therapies such as platinum-based chemotherapy. Once patients experience disease progression on platinum-based chemotherapy and maintenance first-line immunotherapy, there are limited treatment options due to the lack of UK reimbursement for internationally recognized options such as ADCs.

A major challenge with the healthcare system’s ability to offer systemic anticancer treatments is the capacity to deliver the treatment. Of the currently approved treatments, cisplatin is the highest burden for cancer treatment units because of the long chair occupancy of 4 to 8 hours per infusion. Additionally, patients treated with chemotherapy tend to have more side effects than those on immunotherapy,^31^ which could lead to more calls to acute oncology services for advice. The current schedule of maintenance immunotherapy every 2 weeks also presents a significant burden for both patients and hospitals.

These capacity issues, alongside budget impact and low cost-effectiveness, are seen as the main challenge to offering innovative treatments in the United Kingdom. Many day units in the National Health Service are already struggling to provide the currently available maintenance therapies for many different tumor types. The impact of innovative treatments on day unit capacity, including pharmacy services, chair space, and pharmacists’, nurses’, and oncologists’ time, creates challenges that need to be addressed. Some centers are better able to offer advanced treatments, which then creates “postal code” inequality.^28^^,^^29^

### Recommendations for implementation of EV-P in the United Kingdom

#### Healthcare system implementation considerations

If EV-P is funded in the United Kingdom, this group recommends leveraging lessons from previous implementation of immunotherapies (Table S1). Guidelines should be created to ensure the effective uptake of this combination treatment, taking into account the considerations of the multidisciplinary team and the existing challenges of the treatment units throughout the country. National Institute for Health and Care Excellence guidelines for EV-P in advanced UC are currently in development, with an expected publication date of July 2025.^32^ EV-P has not yet been added to UK chemotherapy protocols.

Clinical centers that were involved in the EV-302 clinical trial will be valuable in providing peer support to other treatment centers as they begin to offer this combination. This group recommends that centers with experience share protocols and that their clinical staff provide advice and support to fellow key clinical staff across different sites, particularly in toxicity monitoring, identification, and management. Specialist pharmacists can play a critical role by sharing protocol documents and local builds on electronic oncology prescribing systems. A therapy management tool on digital medical apps such as ONCOassist^33^ has been created for EV-P for quick references to national and international management guidelines to assist in the identification of side effects and management of toxicity. The group recommends that a centralized protocol team from trial sites create the protocols for dissemination across different centers. Key clinical trial sites would ideally provide advice and support for centers that have more than 10 patients with bladder cancer per year as the first phase of the implementation. However, once the initial phase is completed successfully, then treatments like EV-P should be rolled out to other centers with support from the experienced network of centers and clinicians.

Referral pathways for the management of AEs grew organically when immunotherapy was first introduced. Although networks are easier to establish in larger academic centers, when EV-P is introduced in the United Kingdom, we encourage smaller centers to collaborate with nearby specialist/regional/supra-regional centers, as referral networks need to be in place when hospitals start offering this combination. In some areas, such referral networks already exist; for example, there are specialist skin multidisciplinary teams at larger centers that smaller centers can link to, but not all centers may have adequate access. There is some concern that without referral pathways in place, acute oncology services at smaller hospitals may not have the experience in identifying and treating some of the side effects of this combination therapy. This could delay appropriate referral for further care.

Skin reactions are a common side effect of immunotherapies. Hence, there has been a lot of experience gained over the last decade in managing this side effect. Dermatology specialist involvement in this area is associated with a lower risk of disruption in oncologic management (either with systemic immunosuppression or immune checkpoint discontinuation; odds ratio 0.03; *P* = .015)^34^ and increased immune checkpoint retrial following interruption with improved PFS and OS.^35^ Furthermore, 4% of dermatologists vs 29% of referring clinicians recommend treatment interruption for dermatologic AEs.^36^ Hence, establishing referral pathways with dermatology is likely to be beneficial on multiple fronts, and these benefits may, logically, be extended to dermotoxic drugs with alternative mechanisms of action.

Additionally, in the United Kingdom, groups such as the UK Acute Oncology Society and the Immuno-Oncology Clinical Network aim to provide service development and clinical guidance, education, and a support network for managing and implementing new oncology treatments.^37^^,^^38^ National Hospital for Neurology and Neurosurgery and University College London Hospitals have a national immunotherapy neurotoxicity multidisciplinary service with access to email advice daily and biweekly online multi-specialty discussion and advice meetings.^39^ These groups can provide additional resources for centers seeking guidance and support.

The referral and multidisciplinary care model needs to be aligned with the care model at different types of hospitals. Many district general hospitals only have consultant specialists, with no foundation year or registrar specialists who can assist in managing patients. Multidisciplinary team members such as nurses, pharmacists, or prescribing pharmacist centers could be engaged for pre-assessment or ongoing monitoring.

Education of bladder cancer clinical nurse specialist teams, acute oncology service nursing teams, pharmacy teams, and clinicians in proactive side effect management and identification will be necessary. This education and training will be essential to ensure that patients who could benefit from therapy are not excluded due to concerns about manageable side effects. Education and training can be provided upon request from the pharmaceutical company, a larger academic center, or an EV-302 trial site.

Attention needs to be paid to patients’ medical histories to uncover possible risk factors for AEs. A suggested pre-initiation checklist for the EV-P combination is shown in Table 1, and suggested patient assessments and checklists prior to and during treatment with EV-P can be found in Table S2. These checklists are meant for advanced clinical practitioners and should be used in conjunction with the Padcev Summary of Product Characteristics, Patient Information leaflet, enfortumab vedotin Patient Booklet from the pharmaceutical company, and Patient Alert Card.^40^^,^^41^ The checklists focus on the following patient subgroups:

**Table 1.** Suggested pre-initiation checklist for the enfortumab vedotin and pembrolizumab combination.

	Things to consider^a^: clinical use is best guided by physician judgement
Prior lines of therapy	Indicated for first-line therapy
Performance status	ECOG performance status 0, 1, and 2 patients are eligible
Meets minimum renal function	15-30 mL/min^b^
Patients for whom the treatment is indicated but were excluded from the EV-302 trial	Hearing loss ≥ grade 2 New York Heart Association class II heart failure Diabetes with HbA1C up to 13.9 mmol/L^c^ Moderate to severe liver dysfunction Neuropathy ≥ grade 1. Patients with ≥grade 2 were excluded.

a Based on inclusion and exclusion criteria for the EV-302 clinical trial.^66^^,^^24^

b The UK summary of product characteristics states that enfortumab vedotin can be used in patients with severe renal impairment (creatinine clearance 15 to < 30 mL/min).^40^ There were no clinically significant differences in pharmacokinetic parameters for enfortumab vedotin in patients with severe renal impairment.^67^^,^^64^ In addition, pembrolizumab may be used without dose adjustment in patients with altered kidney function.^68^^,^^64^

c Uncontrolled diabetes in EV-302 was defined as hemoglobin A1c (HbA1c) ≥ 10 mmol/L or HbA1c 8.6 to 10 mmol/L with associated diabetes symptoms (polyuria or polydipsia) that are not otherwise explained.^1^

Abbreviations: ECOG, Eastern Cooperative Oncology Group; EGFR, epidermal growth factor receptor.

- Prior history of autoimmune disorders. Thirty-nine percent to 50% of those with pre-existing/prior autoimmune disease will experience an exacerbation with ICIs.^42^
- Possible subclinical autoimmune disease.
- A history of skin conditions such as rashes, or previous cutaneous reactions to systemic anticancer therapies, may be reactivated by immunotherapy—even after years of remission.^43^ However, prior skin toxicity to one immunotherapy agent does not necessarily predict recurrence with another agent.
- Pre-existing neuropathy.
- Ocular disorders. A baseline past ophthalmic history from the community optometrist is helpful for evaluating ocular AEs from immunotherapy.
- Hyperglycemia.

This group recommended using a checklist for enfortumab vedotin adverse event management, as is done for various other systemic anticancer treatments. Until there is more clinical experience, decision-making on when to stop therapy for lower-grade, chronic toxicity will be a challenge. The checklist could include red flags and key questions to ask to uncover under-reported or milder events before they become higher grade. Close follow-up and monitoring are needed to support patients on therapy. Diagnosis of AEs may sometimes require collaborating with appropriate specialties. The checklist should include when to refer to another specialty or specialist for the management of AEs. The prescribing team will set the frequency of review, which may change over time or for individual patients. At least one team member should review the patient at every cycle, though it may not necessarily be the oncologist.

Some of the tests conducted prior to each treatment in the trial are not performed routinely in clinical practice (eg, lipase, B-type natriuretic peptide), and there is variability between centers based on local practice and availability due to different care models at different district general hospitals. In some centers, oncologists commonly treat bladder cancer, while in others, general oncologists treat a wider variety of tumors. Different approaches are needed for these different models.

A suggested checklist for ongoing monitoring after cessation of EV-P is shown in Table S2. Because EV-P is indicated as first-line therapy, there is a chance of progression. Hence, monitoring post-cessation of treatment is necessary. After progression on EV-P, platinum-based chemotherapy may be an option for treatment, depending on toxicity.

### Management of AEs

#### AEs associated with EV-P

The safety results in the EV-302 trial were consistent with the known adverse reactions of the respective study treatments and/or underlying disease, preexisting comorbidities, and advanced age of the study population. In the EV-P group, the most common AEs were peripheral sensory neuropathy, pruritus, and alopecia, occurring in 50.0%, 39.8%, and 33.2% of the study population, respectively. The most frequent grade 3 or higher AEs were maculopapular rash, hyperglycemia, and neutropenia, in 7.7%, 5.0%, and 4.8% of the study population.^24^

Grade 3 or higher AEs of special interest that were previously associated with enfortumab vedotin included severe skin reactions (15.5%), peripheral neuropathy (6.8%), and hyperglycemia (6.1%). Grade 3 or higher AEs of special interest that were previously associated with pembrolizumab included severe skin reactions (11.8%), pneumonitis (3.6%), and hepatitis (1.8%). Most of these AEs were manageable with dose modifications.^24^

#### Managing AEs with dose modifications

Dose modifications, including reductions and interruptions, are recommended to manage EV-P-related AEs. In the EV-302 clinical trial, dose reductions due to treatment-related AEs occurred in 40.7% of study participants.^24^ In this study, participants assigned to the EV-P arm received enfortumab vedotin as an intravenous infusion (at a dose of 1.25 mg per kilogram of body weight with a maximum of 125 mg per dose) on days 1 and 8 and pembrolizumab as an intravenous infusion (at a dose of 200 mg) after the enfortumab vedotin infusion on day 1 of each 3-week cycle.

A post hoc exploratory analysis across enfortumab vedotin monotherapy trials EV-101, EV-201, and EV-301 demonstrated that PFS and OS improvements were seen across all therapy exposure quartiles, inclusive of dose modifications.^44^ Greater enfortumab vedotin exposure in the first 2 cycles was associated with a higher ORR, consistent with a dose–response effect. However, lower enfortumab vedotin exposure was associated with a lower risk of enfortumab vedotin-related grade 3 or higher rash or skin reactions, grade 2 or higher peripheral neuropathy, and grade 3 or higher hyperglycemia. These results demonstrate that the starting dose of enfortumab vedotin of 1.25 mg/kg on days 1, 8, and 15 of every 28-day cycle helps ensure patients have an effective dose intensity; however, dose modifications are effective for managing enfortumab vedotin-related AEs and should be used as clinically indicated. AEs and recommendations for dose modifications and other management are summarized in Table 2 and described in detail in the sections below. The recommended dose modification schedule for enfortumab is shown in Table S3 and is also available in the Summary of Product Characteristics.^40^

**Table 2.** Adverse event management and dose modifications.

Adverse reaction^40^ ^,^ ^43^	Severity^40^ ^,^ ^43^	Dose modification and other actions^40^ ^,^ ^43^
Skin reactions	Grade 1 Macules/papules covering < 10% BSA with or without symptoms (eg, pruritus, burning sensation/pain, skin tightness)	Closely monitor and continue at the same dose level with supportive care as clinically indicated. Fragrance-free moisturizer and soap substitutes should be used over all body surfaces. Strong topical corticosteroids can be used on affected skin areas. Antihistamines may also be used.
	Grade 2 Macules/papules covering 10%-30% BSA with or without symptoms; limiting instrumental ADL	Closely monitor and continue at the same dose level with clinically indicated supportive care as outlined above. Very strong topical corticosteroids can be used on affected skin areas on the torso and limbs. Clinically reassess after 4 weeks.
	For persistent or recurrent grade 2 skin reactions. Macules/papules covering 10%-30% BSA with symptoms; limiting instrumental ADL	Consider withholding until grade ≤ 1, then resume treatment at the same dose level or dose reduce by one dose level and resume pembrolizumab. Consider specialist referral.
	Grade 2 worsening Grade 2 with fever Grade 3 skin reactions Macules/papules covering > 30% BSA with moderate or severe symptoms; limiting self-care ADL	Hold both agents if rapid onset or worsening of symptoms. Oral corticosteroids (1-2 mg/kg daily) while holding both drugs until complete or partial resolution to grade ≤ 1. Specialist referral, consider skin biopsy to assist with diagnosis. Withhold until grade ≤ 1, then resume enfortumab vedotin at the same dose level or dose reduce by one dose level. Consider reintroduction of pembrolizumab depending upon the severity of skin reaction.
	Suspected SJS or TEN Bullous pemphigoid blisters covering < 10% BSA	Immediately withhold, consult a dermatologist to confirm the diagnosis. Referral to specialist regional TEN Center. If not SJS/TEN, see grade 2-4 skin reactions.
	Confirmed SJS or TEN Grade 4 or recurrent grade 3 skin reactions Grade 4 reactions are erythema covering > 90% (erythroderma) BSA with associated fluid or electrolyte abnormalities; ICU care indicated	Permanently discontinue. Consider inpatient and specialist management. IV corticosteroids (methylprednisolone 1 mg/kg^-1^ daily or equivalent) Skin biopsy may assist with diagnosis.
Peripheral neuropathy	Grade 1	Consider proactive dose reduction or dose hold for enfortumab vedotin Consider holding pembrolizumab (low threshold to hold), monitor symptoms closely for a week Consider supportive treatment with medications for nerve pain.
	Grade 2	Withhold until grade ≤ 1, then resume treatment at the same dose level (if first occurrence). For a recurrence, withhold until grade ≤ 1, then resume treatment reduced by one dose level. Consider investigations for immune-mediated cause. Consider supportive treatment with medications for nerve pain. Consider physical or occupational therapy. Consider nerve conduction studies in cases of uncertain etiology. Consider neurology input for atypical grade 2 reactions.
	Grade ≥ 3	Permanently discontinue. Specialist referral.
Hyperglycemia	Grade 1	Continue enfortumab vedotin and pembrolizumab with close clinical follow-up and laboratory evaluation. Initiate insulin therapy/anti-hyperglycemic as clinically indicated.
	Blood glucose > 250 mg/dL	Withhold until elevated blood glucose has improved to ≤ 250 mg/dL, then resume treatment at the same dose level. Continue pembrolizumab. Initiate insulin therapy/anti-hyperglycemic as clinically indicated.
	Grade ≥ 3	Permanently discontinue. Consider urgent endocrine referral. Inpatient admission for management of concern for developing diabetic ketoacidosis, symptomatic patients regardless of diabetes type, new onset of type 1 diabetes mellitus, unable to see endocrinology.
Pneumonitis/ILD	Grade 2	Hold both agents. Consider referral to respiratory specialist Exclude typical and atypical infection; start antibiotics as per local guidelines if there is suspected infection Administer corticosteroids (initial dose of 1-2 mg/kg of prednisone or equivalent, followed by taper) with gastric protehction Following corticosteroid taper, withhold until grade ≤ 1, then resume treatment at the same dose level or consider dose reduction by one dose level.
	Grade ≥ 3, recurrent grade 2	Permanently discontinue Administer corticosteroids (initial dose of 1-2 mg/kg prednisone or equivalent, followed by taper). Urgent referral to respiratory specialist/ILD team. Hospitalize; consider ICU care.
Other non-hematologic toxicity	Grade 2	For *ocular toxicity*: hold EV-P until grade ≤ 1 then resume at the same dose level. Recommend moisturizing eyedrops as a preventative treatment. Avoid using contact lenses if possible. For *vomiting/diarrhea/colitis:* hold pembrolizumab, continue enfortumab vedotin at the same dose. Use symptomatic management with a low fiber diet and fluids Consider corticosteroids with gastric protection if suspected to be immune-related (initial dose of 1-2 mg/kg prednisone or equivalent) followed by taper Reintroduce pembrolizumab following taper if diarrhea improves ≤ grade 1
	Grade 3	Withhold until grade ≤ 1, then resume treatment at the same dose level or consider dose reduction by one dose level.
	Recurrent grade 3	*Vomiting/diarrhea/colitis:* Permanently discontinue pembrolizumab. Consider corticosteroids if suspected to be immune-related (initial dose of 1-2 mg/kg prednisone or equivalent) followed by taper For persistent immune-mediated diarrhea, consider IV corticosteroids or infliximab. Hospitalize if indicated.
	Grade 4	Permanently discontinue.
Hematologic toxicity	Grade 3 or grade 2 thrombocytopenia	Withhold until grade ≤ 1, then resume treatment at the same dose level or consider dose reduction by one dose level.
	Grade 4	Withhold until grade ≤ 1, then reduce dose by one dose level or discontinue treatment.

#### Skin reactions

Rates of skin reactions, including fatal events, occurred at a higher rate when enfortumab vedotin was given in combination with pembrolizumab compared to either agent alone.^40^^,^^45^^,^^46^ In the pooled safety dataset from the EV-302 trial of EV-P in the first-line advanced UC setting and the EV-103 trial of the combination in first- and second-line advanced UC, skin reactions of all grades occurred in 70% of patients.^40^^,^^43^ The majority of the skin reactions that occurred with the combination therapy were macular, papular, or maculopapular rash.^40^^,^^43^ Grade 3 or 4 skin reactions occurred in 16% and 1% of patients, respectively. A fatal reaction of bullous dermatitis occurred in one patient (0.2%).^40^^,^^47^ Severe cutaneous adverse reactions such as Stevens–Johnson syndrome/toxic epidermal necrolysis have occurred with both EV-P as monotherapies.^48^^,^^49^ Examples of pictures of skin reactions associated with ICIs can be found in Kawsar et al.^50^

**Figure 4.**
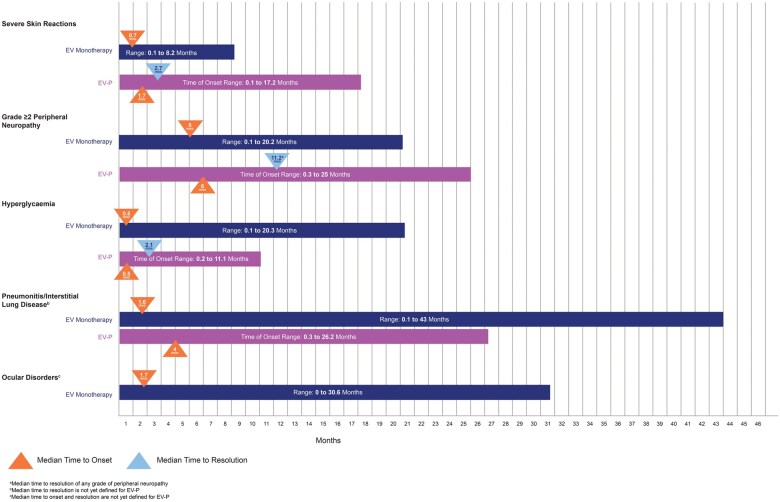
Median time to onset and resolution of adverse events with enfortumab vedotin and pembrolizumab.^39^^,^^44^ Median time to resolution is depicted as the overall time to resolution (median time to onset of the adverse event plus the median time to resolution from onset). Abbreviation: EV-P, enfortumab vedotin and pembrolizumab.

The median time of onset for grade 3 or 4 skin reactions was 1.7 months (range, 0.1-17.2 months); notably, events occurred as early as the first cycle (Figure 4).^40^ Of 391 patients who experienced a skin reaction and had data regarding resolution, 59% had complete resolution. Of 159 patients with an ongoing skin reaction, 27% were grade 2 or higher at last follow-up. Long-term follow-up data (median 4 years) from patients who were ineligible for cisplatin in the EV-103 trial demonstrated that 90% of those experiencing a skin reaction had improvement or resolution of symptoms at the last follow-up.^40^^,^^47^^,^^51–53^ The median time to resolution was 1.0 months.^45^

Currently, oncologists follow guidelines from dermatology for managing typical or low-grade dermatologic toxicities from immunotherapies.^53^^,^^54^ Guidance for the management of dermatologic AEs should focus on the management and recognition of “red flag” symptoms that should prompt immediate referral to an expert dermatologist. Tools for healthcare providers should include education on “red flag” symptoms for immediate referral,^53^ including:

- Skin pain.
- Fever/hypothermia.
- Pustules.
- Blisters.
- Desquamation/erosions.
- Mucosal membrane involvement.
- Target lesions.
- Purpuric lesions.
- Facial edema.
- Lymphadenopathy (new/persistent).

Other reasons for referral include diagnostic uncertainty, grade 2 lesions that are not responding to treatment, or grade 3 reactions, even if the patient appears to be managing well. Body surface area does not always correlate with severity; hence, a dermatologic assessment can help to determine if drug withholding is necessary. If photos are to be sent for a virtual consult, patients should be instructed to follow guidelines on taking photos for such consults.^55^

There are few dermatologists who specialize in skin toxicity to cancer therapies. On-call and acute cover varies significantly between hospitals, with many centers having no on-call cover at all. The risk of skin reactions with EV-P is significantly higher than that observed with immunotherapy alone. Severe (grade 3 or 4) skin reactions occurred in 17% of patients: 16% of patients had grade 3 skin reactions, and 1% of patients had grade 4 reactions.^40^ Based on the previous UK experience with immunotherapy, staff at hospitals should be trained to identify skin toxicities that need to be referred vs those that can be handled by the oncology team. ONCOassist may also be used to help guide management of skin toxicity.^33^

Oncology team members should refer to published guidelines on the recognition and management of skin toxicity associated with immunotherapies.^53^^,^^54^ Differentiating between enfortumab vedotin- and pembrolizumab-related skin toxicities can be challenging.^43^^,^^56^ Sometimes biopsy may be indicated to help differentiate.

All patients should be educated on general skin care prior to starting treatment. This should include advice on emollient use, sun protection, and appropriate soap substitutes. Patient information leaflets and education should also be used to highlight the skin signs and symptoms that should alert patients when and how to contact their team for further advice.^43^^,^^56^

#### Peripheral neuropathy

Peripheral neuropathy occurred more frequently and was more severe when enfortumab vedotin was given in combination with pembrolizumab compared to either agent alone.^43^^,^^46^^,^^47^ Peripheral neuropathy is an anticipated side effect associated with monomethyl auristatin E-containing ADCs and is a cumulative AE with symptoms potentially developing as the duration of treatment lengthens.^43^^,^^57^ This is a length-dependent or glove and stocking distribution, most commonly with painful sensory neuropathy, but mild length-dependent motor weakness may occur in some people. Positive sensory phenomena such as pins and needles, hypersensitivity, and spontaneous electric shocks are often described, but clumsiness and unsteadiness when walking may also occur due to proprioceptive loss.^58^

Immune-mediated neuropathies have been known to occur rarely with pembrolizumab.^54^ Immune-related neuropathy with pembrolizumab is most commonly a sub-acute onset, non-length-dependent polyradiculoneuritis. Patients describe patchy but diffuse, painful positive sensations. Walking and function are limited by a proximal and distal pattern of weakness. This progresses more rapidly than EV-related neuropathy and is not closely related to dosing or timing of immunotherapy but typically occurs within 3 to 6 months of exposure. Prompt treatment with high-dose corticosteroids and discontinuation of immunotherapy is required, and if managed appropriately, the outcome can be good with minimal long-term disability. Delayed recognition and treatment will result in irreversible deficits.^59^

Prompt distinguishment of neuritis secondary to immunotherapy from the more common length-dependent sensory neuropathy due to enfortumab vedotin is particularly important, as management of the 2 conditions differs. Early neurology input improves long-term outcome and survival in patients with immune-related neurological toxicity, and integrated specialist care is essential.^60^ A system for prompt neurological clinical opinion should be set up: many regions are developing models to facilitate prompt review of immunotherapy-related neurotoxicities with a growing awareness of this need.

In the pooled safety dataset from the EV-302 and EV-103 trials of EV-P, peripheral neuropathy was the second most common side effect, occurring in 67% of patients, with grade 3 events occurring in 7% of patients.^40^^,^^43^ Peripheral neuropathy was the most frequent reason for enfortumab vedotin discontinuation^40^^,^^43^; among those who discontinued the medication, 15% in the EV-302 trial and 20% in the EV-103 trial did so because of peripheral neuropathy.^40^ The onset of grade 2 or higher peripheral neuropathy generally occurred later in the treatment course, with a median time of onset of 6 months (range, 0.3-25 months) (Figure 4).^43^^,^^46^^,^^47^^,^^56^

Of the 373 patients who experienced neuropathy and had data regarding resolution, 13% had complete resolution. Eighty-seven percent had residual neuropathy at last follow-up, and 45% had grade ≥ 2 neuropathy.^47^ Long-term data from patients who were ineligible for cisplatin in the EV-103 trial, at a median follow-up of 4 years, demonstrated that nearly 70% of those who had treatment-related peripheral neuropathy had improvement or resolution of their symptoms at their last follow-up.^45^ The median time to resolution of any-grade peripheral neuropathy was 5.2 months.^45^

Careful clinical phenotyping of the neuropathy presentation is required to identify those individuals in whom dose reduction and conservative management are helpful vs those who require immunosuppressive treatment and pembrolizumab discontinuation. Prompt neurology specialist opinion should be sought for any individual with non-length-dependent features.

#### Hyperglycemia

Hyperglycemia and diabetic ketoacidosis, including fatal events, occurred in patients treated with enfortumab vedotin and enfortumab vedotin in combination with pembrolizumab regardless of pre-existing diabetes mellitus.^43^^,^^61^^,^^62^ Although the pathophysiology of enfortumab vedotin-induced hyperglycemia is not well understood, it is manageable and can be resolved. In clinical trials with enfortumab vedotin monotherapy, 17% of patients developed hyperglycemia of any grade, while discontinuation due to hyperglycemia was limited to less than 1% of patients. In the EV-302 trial, hyperglycemia of any grade occurred in 10.9% of patients, compared with 14% with enfortumab vedotin monotherapy and 0.2% with pembrolizumab monotherapy; grade 3 or higher hyperglycemia occurred in 5.0% of patients.^24^^,^^40^^,^^43^^,^^47^

Hyperglycemia presents early, with a median onset time of approximately 2 weeks with both enfortumab vedotin as monotherapy and in combination with pembrolizumab (Figure 4).^40^^,^^43^^,^^47^ Long-term data from patients who were ineligible for cisplatin in the EV-103 trial demonstrated that at a median follow-up time of 4 years, all patients who experienced treatment-related hyperglycemia had improvement or resolution of their hyperglycemia at their last follow-up. The median time to resolution was 1.6 months.^45^ In this cohort, hyperglycemia occurred more frequently in patients with a body mass index of ≥ 30 kg/m^2^ or with baseline hyperglycemia or diabetes mellitus, a trend also observed in a study evaluating enfortumab vedotin monotherapy.^45^^,^^61^

In practice, testing for hyperglycemia has probably been under-performed for many oncology therapies and should become more routine. This group recommends that guidelines from the Joint British Diabetes Societies for Inpatient Care and the UK Chemotherapy Board on hemoglobin A1C and blood glucose monitoring should be followed.^63^

#### Pneumonitis/interstitial lung disease

Pneumonitis/interstitial lung disease (ILD), including severe, life-threatening, or fatal events, occurred in patients treated with both EV-P as monotherapies and occurred at higher rates when given as combination therapy.^46^^,^^47^^,^^50–52^ In the pooled safety population, pneumonitis occurred in 10% of patients treated with EV-P. Grade 3 or higher pneumonitis occurred in 4% of patients and was fatal in 2 patients (0.4%). The median time to the onset of any grade of pneumonitis was 4 months (range, 0.3-26 months) (Figure 4).^43^^,^^46^^,^^47^

There is a high level of awareness of how to manage pneumonitis/ILD within the UK clinical community. As a result, good education on recognizing pneumonitis/ILD is already available such as monitoring ILD/pneumonitis through regular clinic reviews, imaging, sputum/blood tests, and assessment of symptoms.

#### Ocular disorders

In clinical trials, the majority of ocular AEs involved the cornea and included events associated with dry eyes.^47^ In patients treated with EV-P in the EV-302 trial, the most common ocular disorder was dry eye, occurring in 18.6% of patients.^24^ It was generally mild, with no events ≥ grade 3. Ocular disorders often presented early, at a median time of 1.6 months.^40^^,^^54^^,^^64^

Although not seen in the clinical trial setting for EV-P, ICI therapy can also be associated with ocular toxicity and with Triple-M overlap syndrome, which is a rare complication with a high mortality rate of 37%.^65^ Triple-M syndrome is a combination of myasthenia (ptosis, diplopia, dysarthria, limb, and neuromuscular respiratory weakness), myocarditis, and myositis. It presents with double vision and limb aches in individuals over 70 years of age treated with PD-L1 or programmed cell death protein 1 inhibitors. A low threshold for screening creatine kinase, troponin, and a clinical assessment for myasthenic weakness is recommended as a baseline assessment for patients who fit this risk profile.

Ocular toxicity is seen with other systemic anticancer agents as well. Trained clinical nurse specialists have been used in one private hospital to conduct ocular assessments before each cycle of therapy for a different ADC, with onward referral to a specialist if concerns are identified. These referral pathways are just starting to form in many centers but are more established in centers that have active clinical trial programs. However, ophthalmologists with this interest and expertise are few.

Patients should be encouraged to see their optometrist on a yearly basis regardless of their other systemic comorbidities. Good care involves having a community optometrist as a key component of a patient’s eye health management. In the context of new AEs, the community optometrist can refer to the local ophthalmologist, who can, in turn, refer to a specialist unit if needed.

## Conclusions and future directions

The last few years have brought significant advancements in the bladder cancer arena, with a number of therapies being licensed in the first-line setting. However, there remains the need for data on the most appropriate sequencing after first-line therapies, as well as longer-term efficacy and safety data.

To support capacity planning and manage chemotherapy workloads in day units, further studies extending the expiry of reconstituted antibody drug conjugates and subcutaneous formulations of immunotherapies would help significantly with the workload and capacity pressures in chemotherapy day units and facilitate home administration. With further lines of treatment now available that have the potential to increase OS, long-term follow-up and early management are essential to enable patients to be able to tolerate further lines of treatment if necessary.

Access to life-extending therapies is a crucial issue of equity for patients with advanced UC. Patients with other tumor types have long benefited from such therapies, and it is essential to address capacity and implementation challenges so that individuals with advanced UC can access similar treatments. Furthermore, by enabling high-quality management of AEs, patients may be able to remain on treatment for longer, thereby improving the duration of clinical benefit.

## Supplementary Material

oyaf220_Supplementary_Data

## Data Availability

No new data were generated or analyzed in this manuscript.
