# Correction to: Astellas article: UK multidisciplinary recommendations on use of combination first-line enfortumab vedotin▼ and pembrolizumab in advanced urothelial carcinoma

**DOI:** 10.1093/oncolo/oyag031

**Published:** 2026-02-17

This is a correction to: Syed Hussain, Aisling Carr, Simon John Crabb, Vikash Dodhia, Louise Fearfield, Jahangeer Mahmood Malik, Maria Lapuente, Davina Lau, Harry Petruskin, Naveed Sarwar, Nicola Jane Smith, Robert Stevenson, Robert Jones, Astellas article: UK multidisciplinary recommendations on use of combination first-line enfortumab vedotin▼ and pembrolizumab in advanced urothelial carcinoma, *The Oncologist*, Volume 30, Issue 10, October 2025, oyaf220, https://doi.org/10.1093/oncolo/oyaf220

The title is emended to read: “Astellas article: UK multidisciplinary recommendations on use of combination first-line enfortumab vedotin▼ and pembrolizumab in advanced urothelial carcinoma” instead of: “UK multidisciplinary recommendations on use of combination first-line enfortumab vedotin ▼ and pembrolizumab in advanced urothelial carcinoma”.

The following has been added before section Background at the beginning of the Abstract: “Prescribing information & ADR reporting: This is an Astellas article. Prescribing information can be accessed at https://dhgd52pup2eiv.cloudfront.net/23ffe42b-4e89-48e1-b8d8-08b726ed94c1/b5d6cdf5-d05a-4bb1-9965-de9a244d39eb/b5d6cdf5-d05a-4bb1-9965-de9a244d39eb_source__v.pdf and the adverse event reporting information can be found on the last page of that link.”

