# Supplementary material for: Astellas article: UK multidisciplinary recommendations on use of combination first-line enfortumab vedotin▼ and pembrolizumab in advanced urothelial carcinoma
Source: Oncologist. 2025 Nov 10;30(10):oyaf220. doi: 10.1093/oncolo/oyaf220 (PMC12574332; doi:10.1093/oncolo/oyaf220)
Supplement: oyaf220_Supplementary_Data [file oyaf220_supplementary_data.zip › Supplementary Materials Tables June 25.docx]

**Supplemental Table S1. Best practice considerations from previous implementation of new immunotherapies.**

| One center holds an endocrinology clinic once a month for patients on immunotherapy. |
| --- |
| Virtual oncology multidisciplinary team meetings. |
| Creation of a small network of specialists to reach out to on an *ad hoc* basis. |
| An e-consult system with expert dermatologists who have a special interest in skin toxicities from immunotherapy and ADCs. These dermatologists can assess some patients through photographs. Some trusts in central London have such a system in place. |
| Initiating treatment at academic centers at first while oncology teams learned and developed best practices among the experienced oncology staff. Once these practices were established after about six months, treatment was then moved to the associated peripheral centers. |
| Introduction of immunotherapy toxicity tool on ONCOassist to aid in identification of side effects and management of IO toxicity. |

**Supplemental Table S2. Suggested patient checklists to be used as pre-assessment/prior to cycle 1, for monitoring during treatment cycles, and upon cessation of treatment with enfortumab vedotin and pembrolizumab.** Depending on the hospital workflow, the pre-assessment checklist can be reviewed by nursing, pharmacy, or the oncologist, and the monitoring checklist for treatment cycles may be reviewed by nursing or pharmacy the day before or the day of chemotherapy day unit (CDU) attendance. Abbreviations: BMI, body mass index; CYP3A4, cytochrome P4503A4; DKA, diabetic ketoacidosis; HbA1c, hemoglobin A1c; IV, intravenous; OTC, over-the-counter; Q21D, every 21 days; PABA, para-aminobenzoic acid; PRN, pro re nata/as needed; SACT, systemic anticancer therapy; SJS, Stephens-Johnson syndrome; SmPC, Summary of Product Characteristics; SPF, sun protective factor; T4, thyroxine; TEN, toxic epidermal necrolysis; TFT, thyroid function test; TSH, thyroid stimulating hormone

| **Pre-assessment/prior to cycle 1 patient checklist** | | |
| --- | --- | --- |
|  | **Risk factors to look out for** | |
| Family planning advice | Contraception advice given | |
| Vascular access | Extravasation risk unknown: treat as vesicant  Consider central line | |
| Clinical presentation/history | Skin   - History of dermatological conditions - Skin reactions to prior systemic anticancer therapies (SACT) - Dry skin - High sun exposure - Prior radiation therapy to any area of the body - Immunosuppression   Neuropathy   - Spinal involvement, nonmalignant spinal disease - Family history of neuropathy   Hyperglycemia   - Pre-existing diabetes or hyperglycemia - BMI ≥ 30 kg/m2 - Use of systemic steroids - Underlying fatty liver disease - Illness/infection   Pneumonitis/ILD   - Prior thoracic radiation - History of pulmonary disease   Ocular toxicity   - Existing dry eyes - Contact lenses use - Eye surgery - Current/recurring eye infection | |
| Medication history | Formal drug-drug interaction studies with enfortumab vedotin have not been conducted.  See section 4.5 and section 5.2 of SmPC for further information. | |
| Test to order for baseline (in addition to local guidelines) | - Full blood count - Liver function - Renal function - TFTs (T4, TSH) - Cortisol - HbA1c - Non-fasting blood glucose - Blood pressure - Baseline lung function - Creatinine kinase - Troponin | |
| Physical assessments | General skin assessment  Existing neuropathy   - Symptom review: Numbness, tingling, or discomfort in hands or feet since starting therapy; if yes, tailor clinical exam accordingly - Pain evaluation if discomfort is described - Functional assessment: Observe patient buttoning or zipping clothing, signing name, walking in a straight line, climbing stairs - Physical assessment: Deep tendon reflexes, light touch, temperature, vibration, proprioception   Ocular health  Lifestyle/diet:   - Diabetes - Breathless on exertion | |
| **Monitoring checklist for treatment cycles** | | |
|  | | **Risk factors to look out for** |
| Tests  Q21D (unless worsening symptoms then would recommend increased frequency) | | - Full blood count - Liver function - Renal function - TFTs (T4, TSH) every 9 weeks - Cortisol - HbA1c - Blood glucose (if indicated) - Lung function (only if clinically indicated) - Troponin - Creatinine kinase |
| Physical assessments  Each visit  Assess patient based on grade of severity in SmPC | | General skin assessment   - Onset varies from days to weeks following drug exposure - Systemic symptoms include fever and flu-like symptoms - Eyes, mucous membranes, and visceral organs may be involved - Secondary infections, including sepsis and viral reactivation, can be major complications   Neurologic assessment   - Symptom review: Numbness, tingling, or discomfort in hands or feet since starting therapy; if yes, tailor clinical exam accordingly - Pain evaluation if discomfort is described - Functional assessment: Observe patient buttoning or zipping clothing, signing name, walking in a straight line, climbing stairs - Physical assessment: Deep tendon reflexes, light touch, temperature, vibration, proprioception   Pneumonitis/ILD   - Monitor for:   - Dyspnea   - Cough   - Chest pain   - Decreased activity tolerance   - Hypoxia   - Non-productive cough   - Unresolved dyspnea   - Interstitial infiltrates on radiologic exams   Blood glucose levels |
| Prevention/counseling | | Patient booklet  Take home medications  Red flags for which to call the hospital   - Signs of infection   Eye health   - Good hygiene around the eyes - Cool compression (dry eye, conjunctivitis) - Warm compression (blepharitis) - Lubricating eye drops and ointment around the eye - Avoid using lubricating eye drops containing active ingredients for other symptom management - Avoid wearing contact lenses   Skin health   - Report rash or any new or changing skin reactions immediately, especially if accompanied by a fever or burning sensation. May include ulcers/sores in mucosal areas. - Use SPF ≥ 30 sunscreen, free of PABA - Avoid hot showers - Stay hydrated - Use mild detergents and skin cleansers - Alcohol-free, fragrance-free hypoallergenic moisturizer - Avoid OTC acne medication where possible   Neuropathy   - Symptoms of new or worsening peripheral neuropathy - Simple tests: picking up a coin, buttoning a shirt, handwriting - Numbness and tingling of hands and feet - Pain to change of temperatures - Muscle weakness in legs: report urgently - Precautions for peripheral neuropathy (fall prevention, using oven mitts, and avoiding very hot/cold water) - General foot care advice: check between toes, get someone else (podiatrist) to cut toenails, comfortable shoes, always wear socks   Hyperglycemia   - Counsel patients on signs and symptoms of hypoglycemia and hyperglycemia - Self-monitoring of blood glucose for existing diabetic patients   DKA clinical presentation   - Elevated glucose levels, excessive thirst, frequent urination, constantly feeling fatigued, confusion, dry skin, dry mouth, fruity odor of breath, shortness of breath, nausea, vomiting, abdominal pain   Pneumonitis/ILD  Monitor for:   - Dyspnea - Cough - Chest pain - Decreased activity tolerance |
| Referrals if patient presentation requires it | | Referral to neurologist if following are suspected:   - Urgent: Myasthenia gravis (muscle weakness, dysphagia, ptosis, vision changes) - Urgent: Guillain-Barre syndrome (muscle weakness with absent or reduced tendon reflexes) - Urgent: Encephalitis (confusion, altered behavior, headaches, seizures) - Neuropathy where there is uncertainty about drug-relationship   Referral to ophthalmologist for:   - Urgent: double vision alongside limb aches (related to immunotherapy side effects)   Referral to dermatology is indicated for skin reactions that:   - Have grade 2 worsening rash with fever - Are persistent/recurrent grade 2 - Exceed 30% of body surface area (Grade 3 or higher) - Involve the mucosa, bullous lesions, or exfoliation, or - Do not respond to a combination of steroids, antihistamines, and dose modifications - Are suspected SJS/TEN cases. Early presentation of SJS/TEN may include erythema, blisters, or erosions of the nasopharynx, oropharynx, eyes, genitalia, or anus mucous membranes   Referral to endocrinologist if DKA is suspected:   - Hold SACT - Inpatient management - Manage as per hospital DKA guidelines   Referral to respiratory specialist if suspected grade 2 pneumonitis or worse:   - Hold both agents - Exclude typical and atypical infection; start antibiotics as per local guidelines if there is suspected infection - Administer corticosteroids (initial dose of 1-2 mg/kg of prednisone or equivalent, followed by taper) with gastric protection - Following corticosteroid taper withhold until Grade ≤ 1, then resume treatment at the same dose level or consider dose reduction by one dose level |
| Dose of SACT | | Are dose modifications required? |
| Consider pre-medications to be prescribed for EV-P (on clinical judgement: the SmPC (1) states that no pre-medication is necessary). | | - IV antihistamine - Oral anti-emetic PRN - Avoid steroid premedication if possible - Prophylactic artificial tears - Prophylactic use of moisturizers - Laxative PRN for constipation - Other |
| **Patient checklist upon cessation of treatment with enfortumab vedotin** | | |
|  | | **Follow-up appointments** |
| Monitoring | | Will the patient go on to another treatment?   - If yes, refer to other treatment protocol - If no, an appointment every 3 months is scheduled (or as per hospital policy)   Advise the patient to call chemotherapy day unit if:   - Worsening of existing side effects from SACT - Unexplained fever - Infection/illness - Chemotherapy day unit contact number was given to the patient |

**Supplemental Table S3. Recommended dose reduction schedule for enfortumab vedotin (1).**

| Starting dose | 1.25 mg/kg up to 125 mg |
| --- | --- |
| First dose reduction | 1.0 mg/kg up to 100 mg |
| Second dose reduction | 0.75 mg/kg up to 75 mg |
| Third dose reduction | 0.5 mg/kg up to 50 mg |

**References**

1. Astellas Pharma Ltd. Padcev 20 mg powder for concentrate for solution for infusion [summary of product characteristics]. Addlestone, UK2024.
